# *Trypanosoma cruzi* P21 Is a Pleiotropic Protein That Is Involved in Parasite Host Cell Invasion and Intracellular Parasitism

**DOI:** 10.1002/mbo3.70154

**Published:** 2025-11-10

**Authors:** Nelsa Paula Inácio Uombe, Teresiama Velikkakam, Anna Clara Azevedo Silveira, Cassiano Costa Rodrigues, Bruna Cristina Borges, Thaise Lara Teixeira, Cecília Luiza Pereira, João Paulo Silva Servato, Normanda Souza Melo, Renato Arruda Mortara, José Franco da Silveira, Claudio Vieira da Silva

**Affiliations:** ^1^ Instituto de Ciências Biomédicas Universidade Federal de Uberlândia Uberlândia Minas Gerais Brazil; ^2^ Escola Superior de desenvolvimento Rural—UEM (Universidade Eduardo Mondlane Vilanculos Moçambique; ^3^ Departamento de Microbiologia, Imunologia e Parasitologia Universidade Federal de São Paulo São Paulo Brazil; ^4^ Universidade de Uberaba Uberaba Minas Gerais Brazil

**Keywords:** cell invasion, CRISPR/Cas9, intracellular multiplication, parasite‐host interaction, *Trypanosoma cruzi*, virulence

## Abstract

We characterized the secreted *Trypanosoma cruzi* P21 protein and hypothesized its role in parasite invasion and multiplication. To investigate the role of *T. cruzi* P21 protein in host‐parasite interactions, specifically focusing on the low‐virulence G strain. P21 knockout parasites were generated using CRISPR/Cas9. Cell invasion, multiplication, egress, and tissue parasitism were assessed in vitro and in vivo, comparing knockout and control parasites. P21 knockout significantly reduced parasite invasion and multiplication in Vero cells. *In vivo*, knockout parasites also showed reduced heart tissue parasitism in infected mice, despite no observable systemic parasitemia. Accordingly, P21 knockout trypomastigote egress was reduced in Vero cells. P21 plays a pleiotropic role in *T. cruzi* infection, differentially impacting parasite biology in the low‐virulent G strain. In the G strain, P21 promotes invasion and persistence, potentially through mechanisms distinct from its role in the Y strain previously described. This highlights its potential as a therapeutic target for Chagas disease, warranting further investigation into strain‐specific functions.

## Graphical abstract

This study reveals the pleiotropic role of *Trypanosoma cruzi* P21 protein in the low‐virulence G strain. P21 knockout significantly impairs parasite invasion, intracellular multiplication, and egress in vitro. *In vivo*, P21 deficiency leads to reduced cardiac tissue parasitism, suggesting its role in establishing and maintaining silent infections. P21 influences parasite persistence and host‐pathogen interactions, highlighting its potential as a therapeutic target with strain‐specific implications.

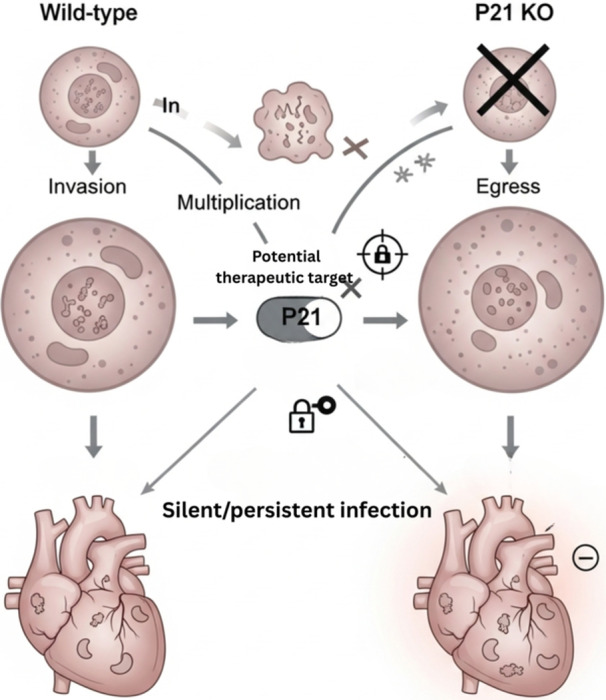

## 1 Introduction

*Trypanosoma cruzi*, the etiological agent of Chagas disease, is a flagellated protozoan of significant public health concern in Latin America. Its complex life cycle involves distinct developmental stages, epimastigote, trypomastigote, and amastigote essential for successful host cell invasion, immune evasion, and the establishment of chronic infections (Brener 1973; de Souza 2007). During cell invasion, infective forms of *T. cruzi* (metacyclic trypomastigotes, bloodstream trypomastigotes, and extracellular amastigotes) employ diverse molecules to interact with host cell components, overcoming the mammalian host's barriers. While the role of P21 in the virulent Y strain has been previously investigated, its function in the low‐virulence G strain and the potential strain‐specific nuances of its pleiotropic activities remain largely unexplored, representing a significant gap in our understanding. This study addresses this gap by characterizing the role of P21 in the G strain and contrasting it with existing knowledge from the Y strain.

*T. cruzi* P21 protein was identified in a comparative gene expression study between the low‐virulence G and virulent CL strains. *In silico* analyses predicted a high probability of protein secretion. Previous studies using recombinant P21 (rP21) demonstrated dose‐dependent adhesion to host cell surfaces, expression across all parasite life stages, secretion, and enhanced cellular invasion by extracellular amastigotes and metacyclic trypomastigotes (da Silva et al. 2009). Subsequently, rP21 was shown to induce macrophage phagocytosis, exhibit chemotactic activity for macrophages and neutrophils, and potentially bind the chemokine receptor CXCR4 (Rodrigues et al. 2012). In a polyester sponge‐induced inflammation model, rP21 recruited immune cells, induced myeloperoxidase and IL‐4 production, and reduced angiogenesis in vitro and in vivo (Martins et al. 2020). This capacity is likely linked to the modulation of actin expression and angiogenesis‐associated genes (Teixeira et al. 2017).

Prior investigations revealed that rP21 treatment reduced parasite load (Y strain) and angiogenesis, while inducing fibrosis in the cardiac tissue of infected mice. Furthermore, rP21 diminished epimastigote growth, inhibited intracellular amastigote replication, and modulated the parasite cell cycle (Teixeira et al. 2019). Corroborating these findings, rP21 decreased *T. cruzi* (Y strain) multiplication in C2C12 myoblasts, associated with increased actin polymerization and IFN‐γ, and elevated IL‐4 expression. During experimental infection (Y strain), mice treated with rP21 exhibited fewer cardiac nests, reduced inflammatory infiltrate, and less fibrosis, correlating with high IFN‐γ expression counterbalanced by elevated IL‐10 levels, consistent with reduced cardiac tissue injury. It was also observed that under stress, such as IFN‐γ exposure, *T. cruzi* upregulated P21 mRNA expression (Teixeira et al. 2015; Teixeira et al. 2022).

Collectively, these data suggest that native *T. cruzi* P21 protein plays a pivotal role in natural infection progression. The observation that recombinant protein induces cell invasion yet reduces intracellular multiplication, coupled with increased native protein expression under stress, implies P21's involvement in a complex mechanism for disease perpetuation. We hypothesize that P21 promotes intracellular parasite persistence and may be upregulated in response to parasite stress. To validate recombinant protein data, we generated P21 knockout parasites using CRISPR/Cas9 gene editing. Initially, we knocked out the P21 gene in the virulent Y strain, demonstrating that P21 knockout metacyclic trypomastigotes exhibited reduced HeLa cell invasion and increased multiplication compared to control parasites (Silveira et al. 2024; also for detailed information on knockout generation, see Silveira et al. 2025).

In this study, we targeted P21 knockout in the G strain, known for its low in vitro virulence and lack of parasitemia induction in experimental in vivo models (Rodrigues et al. 2012). Our strategy involved analyzing various aspects of host‐pathogen interaction in vitro and in vivo using tissue culture‐derived trypomastigotes (TCT) from P21 knockout G strain parasites compared to control (Cas9) parasites. We assessed the protein's effect on invasion, multiplication, and egress in vitro in Vero cell line. *In vivo*, we evaluated systemic parasitemia and inflammatory scores in heart tissue of C57BL/6 mice infected with P21 knockout G strain TCT compared to control parasites. This study aims to verify the strain‐specific and pleiotropic roles of P21 in *T. cruzi* infection, contributing novel insights into the parasite's biology and potential therapeutic interventions.

## 2 Materials and Methods

### 2.1 Parasites and Cell Cultures

Epimastigotes from the G strain were cultured at 28°C in liver infusion tryptose (LIT) medium supplemented with 20% fetal bovine serum (FBS; Invitrogen). Metacyclic forms were obtained by maintaining epimastigotes in LIT for 14 days, and metacyclic trypomastigotes were purified using established protocols (Teixeira and Yoshida 1986). Vero and C2C12 cells (obtained from Instituto Adolfo Lutz, São Paulo, SP, Brazil) were cultured in Dulbecco's minimal essential medium (DMEM) (Sigma Chemical Co.) supplemented with 10% FBS (Cultilab), 10 μg/mL streptomycin, 100 U/mL penicillin, and 40 μg/mL gentamicin at 37°C in a 5% CO2 humidified atmosphere. Stationary phase epimastigote cultures containing metacyclic trypomastigotes from Cas9 and TcP21‐/‐ (G strain) were used to infect Vero and C2C12 cells, generating tissue culture‐derived trypomastigotes (TCT) for in vitro and in vivo experiments. For growth curves, epimastigote forms of the G Cas9 and TcP21‐/‐ strains were cultured at a density of 1 × 10^7 parasites per mL. Parasites were harvested and counted using a Neubauer chamber every 7 days until day 12.

### 2.2 Animals and Ethics

Six‐to‐8‐week‐old male C57BL/6 INFγ knockout mice (15 animals) were housed under standard conditions with a 12‐h light‐dark cycle at 25°C, with food and water ad libitum. Animal care and procedures adhered to the guidelines of the Ethics Committee for the Use of Animals (CEUA). Euthanasia was performed following international welfare standards as per the American Veterinary Medical Association Guidelines. The study was approved by CEUA‐UFU, protocol number: 23117.077543/2022‐27.

### 2.3 Generation of P21 Knockout Parasites

Early‐log phase epimastigotes were transfected with Cas9/pTREX‐n (Addgene Plasmid #68708) (Lander et al. 2015). Selection was performed with G418 (250 μg/mL) 24 h posttransfection, and GFP‐positive parasites were sorted 15 days posttransfection using BD FACSARIA II. SgRNA sequences were designed with EuPatGDT (Peng and Tarleton 2015). DNA templates for sgRNA in vitro transcription were generated by PCR. sgRNAs were transcribed in vitro using the MEGAShortscript T7 kit (Thermo Fisher Scientific). Donor DNA for homologous recombination was produced by PCR using 100 bp ultramers primers. For transfection, 1 × 10^7^ early‐log phase Cas9‐GFP expressing epimastigotes were electroporated with sgRNAs and donor DNA. CRISPR mutant cell lines were maintained under selection with G418, blasticidin, and hygromycin. Genomic DNA was extracted from Wild Type (WT), Cas9‐GFP (Cas9), and knockout lineages (TcP21‐/‐) and analyzed by PCR. The detailed protocol for the generation of these P21 knockout parasites has been previously described in Silveira et al. 2025.

### 2.4 Host Cell Invasion, Intracellular Multiplication, and Egress Assays

Vero cell invasion assays were performed in 24‐well plates containing coverslips. TCT suspensions (MOI: 10:1) were added, and the plates were incubated for 2 h. After incubation, cells were washed, Bouin's fixed, and Giemsa stained. The number of internalized parasites was counted in 300 total cells. For multiplication assays, after invasion, plates were washed, and the medium was replaced. Cells were collected at 24‐, 48‐, and 72‐h postinfection, and DNA was extracted. Experiments were performed in triplicate with three independent biological replicates. For egress assays, following host cell invasion, plates were washed, medium replaced, and incubated. After 72 h, the number of parasites in the supernatant was determined by counting trypomastigote and amastigote forms in a Neubauer chamber for 10 days (240 h) postinfection.

### 2.5 In Vivo Infection

INFγ−/− C57BL/6 mice were randomized into three groups: uninfected, infected with Cas9 parasites (control), and infected with TcP21−/− parasites. Animals were infected intraperitoneally with 10^5^ TCT/mL. Systemic parasitemia was determined from day 3 postinfection. On day 15, animals were euthanized, and hearts were collected for analysis.

### 2.6 Parasite Load Determined by qPCR

Hearts were weighed, stored, macerated, and incubated with NLB buffer, SDS, and PK solution. NaCl buffer was added, and samples were vortexed and placed on ice. Supernatant was collected, ethanol was added, and samples were centrifuged. The pellet was resuspended, and DNA was quantified and analyzed by qPCR. The standard curve was obtained using serial dilutions of 100 ng of DNA extracted from epimastigotes of G strain, with a limit of 0.0001 fg, as proposed by Diaz et al. (1992) and modified by Tavares de Oliveira et al. (2020). Positive, negative, and reagent internal controls were used in all qPCR reactions. The pair of primers used is shown in Table S1. These procedures were also used to quantify parasite DNA in the in vitro multiplication assays.

### 2.7 Inflammatory Score

Heart samples were fixed, processed, and embedded in paraffin. Sections were stained with hematoxylin and eosin (HE). Amastigote nests, inflammatory infiltrate, and tissue damage were evaluated under light microscopy and scored by intensity: (−) absent, (+) mild, (++) moderate, (+ + +) intense as described by da Silva et al. (2018).

### 2.8 Statistical Analysis

Data were presented as mean ± SEM. Normal distribution was checked using the Shapiro‐Wilk test. Significant differences were determined by t‐test and Mann‐Whitney test. Two‐way ANOVA with Bonferroni's or Sidak's test was used for some data. Analyses were performed using GraphPad Prism software. Outlier analysis was performed using Grubbs' test where applicable, but no data points were excluded.

## 3 Results

### 3.1 Knockout of P21 Affected Cell Invasion, Multiplication, and Tissue Parasitism by TCT From *T. cruzi* G Strain

**Figure 1:**
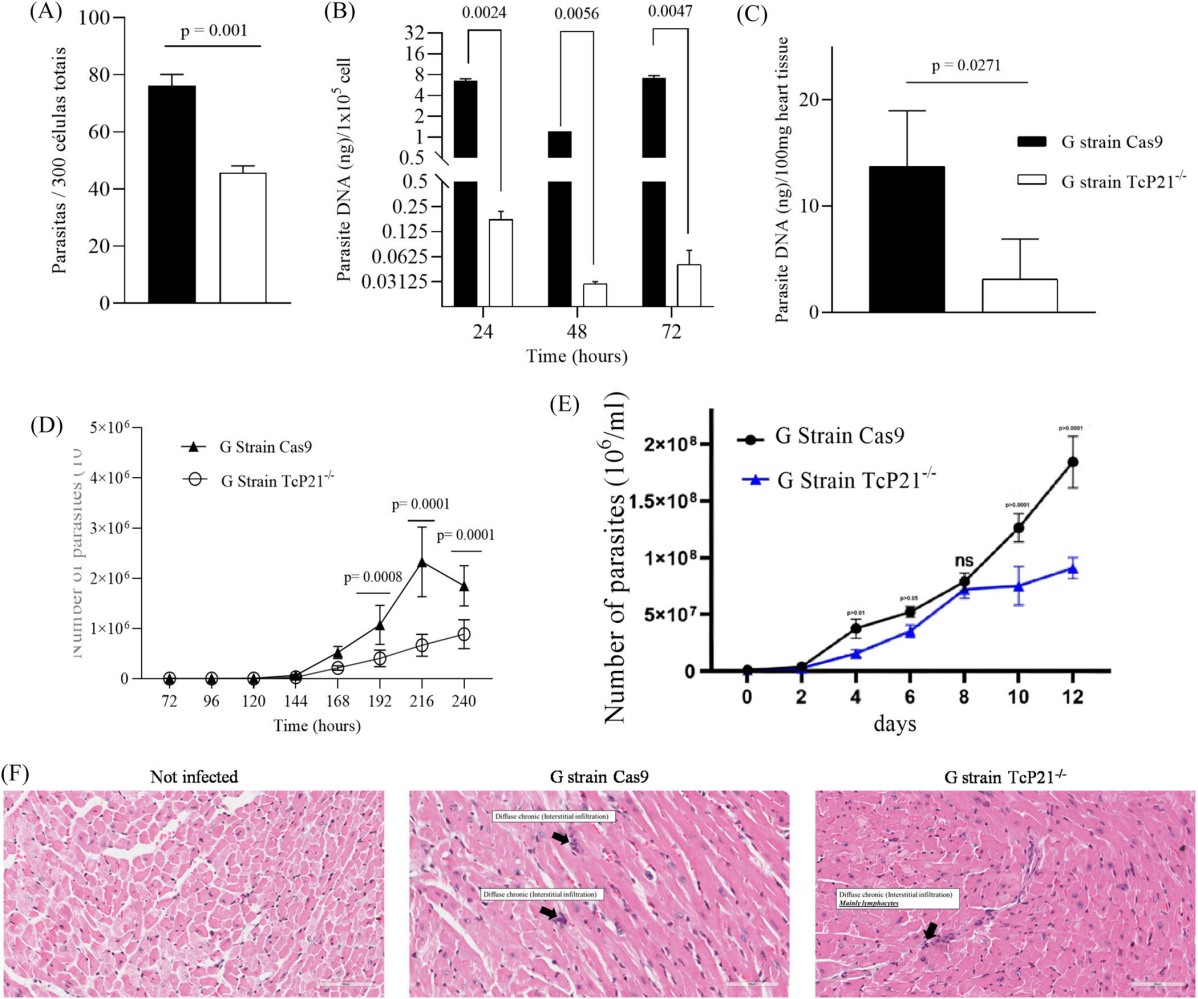
Cell invasion, intracellular multiplication, and tissue parasitism of *T. cruzi* G strain TcP21‐/‐ and Cas9 in the Vero cell line. (A) Vero cell invasion—number of parasites per 300 total cells. (B) Vero cell parasitism determined by the amount of parasite DNA amplified by qPCR. (C) Cardiac parasitism determined by the amount of parasite DNA amplified by qPCR. (D) Egress of trypomastigotes from Vero cells, counted in the supernatant over 10 days. (E) Growth curves of Cas9 and TcP21−/− epimastigotes during 12 days of culture. (F) Representative images of cardiac tissue stained with hematoxylin and eosin. Histopathological alterations are indicated by black arrows.

The successful generation and comprehensive characterization of P21 knockout parasites from G strain validation, have been previously detailed (Silveira et al. 2025). Here, we addressed P21 knockout impact on cell invasion, multiplication, egress, and tissue parasitism in the low‐virulence G strain. We used TCT as the infective form and Vero cell as host cell line. Our results corroborated previous data using rP21 indicating that P21 upregulates parasite cell invasion by *T. cruzi* infective forms. Vero cells infected with TcP21−/− parasites showed significantly lower infection rates compared to cells infected with Cas9 parasites (*p* = 0.001) (Figure 1A).

To assess P21 knockout impact on intracellular multiplication, we allowed Cas9 and TcP21−/− parasites to invade Vero cells for 2 h. qPCR quantification of parasite DNA revealed that Vero cells infected with TcP21−/− parasites had lower parasite DNA content compared to cells infected with Cas9 parasites at 24‐,48‐, and 72‐h postinfection (*p* = 0.0047) (Figure 1B). Trypomastigote egress from Vero cells was significantly lower for G strain TcP21−/− parasites than Cas9 from 168 to 240 h postinfection (Figure 1D).

Previously, we observed that extracellular amastigotes from G strain only established patent infection in IFN‐γ knockout mice (Rodrigues et al. 2012). We used these animals to investigate P21 knockout impact in vivo using TCT from G strain. Surprisingly, neither G strain TcP21−/− nor Cas9 parasite‐infected mice exhibited systemic parasitemia, even in IFN‐γ knockout mice (data not shown), suggesting susceptibility might be restricted to the extracellular amastigote infective form. However, qPCR analysis of heart tissue revealed that animals infected with G strain TcP21−/− showed a significantly reduced parasite load compared to those infected with Cas9 parasites (*p* = 0.0271) (Figure 1C). Histopathological analysis of heart tissue from mice infected with G strain TcP21−/− and Cas9 parasites revealed only mild to moderate alterations compared to control (Figure 1F; Table 1). Consistent with these findings, P21‐deficient epimastigotes of the G strain also exhibited reduced growth compared to control parasites, statistically significant at 4, 6, 10, and 12 days of culture (Figure 1E).

**Table 1:** Qualitative analyses of heart tissues from INF*γ* knockout mice (C57/BL6) at 15 days postinfection with TCT of *T. cruzi* G strain.

Histological criteria	Noninfected (*n* = 3)	G strain Cas9 (*n* = 6)	G strain TcP21‐/‐ (*n* = 6)
Inflammatory response	None	Low/Mild	Low/Mild
Neutrophilic leukocytes	−	+	+
Eosinophils	−	−	−
Macrophages	−	+	+
Lymphocytes	−	++	++
Plasma cells	−	+	+
Giant foreign body cells	−	−	−
Tissue damage	−	+	+
Hydropic degeneration	−	++	++
Necrotic tissue	−	−	−
Apoptotic bodies	−	−	−
Edema	−	+	+
Fibroblast	−	+	+
Fibrosis	−	+	+
Adipocyte	−	−	−
Epicardium calcification	−	−	−
Amastigotes nests	−	+	+

## 4 Discussion

Our prior results with recombinant P21 suggested its involvement in invasion and multiplication processes of parasite infective forms. To confirm this, we initially verified P21 knockout impact on host cell invasion and multiplication by Y strain metacyclic trypomastigotes in HeLa cells. The results corroborated our hypothesis, with knockout impairing parasite host cell invasion and inducing parasite multiplication at 72 h postinfection (Silveira et al. 2024).

Here, we addressed P21 knockout impact on cell invasion, multiplication, egress, and tissue parasitism of the low virulent G strain. Using TCT as the infective form and Vero cell line, our results corroborated previous rP21 data that P21 upregulates parasite cell invasion by *T. cruzi* infective forms.

Considering multiplication rate, G strain knockout parasite multiplication ability was compromised in Vero cells. qPCR data revealed lower multiplication rates in Vero cells for TcP21−/− parasites. These results contrast with those from virulent Y strain infections (Silveira et al. 2024; Teixeira et al. 2022). We hypothesize that in the context of the low‐virulent G strain, P21 might be involved in biological processes supporting parasite persistence within the mammalian host. P21 may exhibit pleiotropic activity, differing across parasite strains. In G strain infection, P21 could maintain a basal cell cycle ensuring perpetuation. Conversely, in Y strain infection, P21 might control multiplication to mitigate parasitism and tissue damage, also contributing to strain survival during infection.

We suggest P21 has a pleiotropic nature, with different activities depending on the strain. In G strain infection, P21 may ensure basal cell cycle for perpetuation, while in Y strain infection, P21 may control multiplication to reduce parasitism and host damage, also ensuring parasite survival. Thus, two biological activities with the same overarching purpose: parasite survival and perpetuation. We also analyzed trypomastigote egress from host cell line. G strain TcP21−/− trypomastigotes egressed from Vero cells in lower numbers than controls throughout the kinetics, consistent with impaired host cell invasion and reduced multiplication.

To verify P21 knockout impact in a complex system, we performed mouse infections. We previously observed that G strain extracellular amastigotes only produced patent infection in IFN‐γ knockout mice (Rodrigues et al. 2012). We used these animals to verify P21 knockout impact in vivo using G strain TCT form. Surprisingly, we observed no parasitemia in either knockout or control parasite infections, suggesting that the susceptibility previously observed might be restricted to extracellular amastigotes infective forms. Consistently, qPCR analysis of heart tissue from TcP21−/− infected animals showed significantly lower parasite load compared to Cas9 infected animals. This in vivo finding, along with in vitro qPCR multiplication data, contrasts with our hypothesis that P21 controls parasite multiplication to maintain intracellular protection and appears to contradict previous findings with Y strain metacyclic trypomastigotes (Teixeira et al. 2022). This discrepancy highlights the strain‐dependent pleiotropic nature of P21. In G strain infection, P21's role and molecular interactions might be for maintaining a regular intracellular amastigote multiplication cycle to perpetuate sub‐patent infection. Histopathological analysis revealed no significant qualitative differences between infection groups compared to uninfected animals. The lack of significant difference in inflammatory scores between groups, despite reduced parasite load in TcP21−/− infected mice, is noteworthy. This contrasts with expectations based on rP21 studies and findings in the Y strain, where P21 appeared to modulate inflammation. In the context of the low‐virulent G strain, P21's role in promoting persistence may be less linked to acute inflammation and more focused on long‐term survival mechanisms. This further supports the hypothesis of strain‐specific pleiotropic functions for P21.

Based on TcP21−/− parasite results from the G strain, we conclude that P21 from the G strain promotes host cell invasion in vitro and sustains in vitro and in vivo parasitism. P21's potential effect on host cardiac tissue parasitism may be related to a mechanism in this non‐virulent strain promoting silent parasite perpetuation in the vertebrate mammalian host. However, it is important to acknowledge the limitations of this study. In vivo experiments were performed in IFNγ knockout mice. This model was chosen because our previous work (Rodrigues et al. 2012) showed that the G strain only establishes patent infection in these immunodeficient animals, allowing us to observe a clear infection phenotype. However, this model may not fully reflect the P21 role in immunocompetent hosts, where the immune response would be different. Furthermore, in vitro studies were limited to the Vero cell line, and further investigation in diverse cell types such as macrophages and cardiomyocytes, and in vivo models is warranted to fully elucidate the pleiotropic roles of P21 across different *T. cruzi* strains and infection contexts. However, it is necessary to acknowledge that the relatively small sample size (*n* = 3–6) for in vivo experiments may limit the statistical power and generalizability of our conclusions. Future studies with larger sample sizes would be beneficial to validate these findings. Nevertheless, this study significantly advances our understanding of P21 function, particularly in the context of the low‐virulence G strain and further establishes P21 as a potential therapeutic target for Chagas disease, emphasizing the need to consider strain‐specific mechanisms in drug development.

## Author Contributions

**Claudio V. da Silva:** conceptualization, supervision, project administration, writing – original draft, writing – review and editing. **José Franco da Silveira:** conceptualization, methodology, funding acquisition, writing – original draft, writing – review and editing. **Renato Arruda Mortara:** conceptualization, methodology, funding acquisition, writing – original draft, writing – review and editing. **Nelsa Paula Inácio Uombe:** investigation, data curation, writing – original draft, writing – review and editing. **Teresiama Velikkakam:** investigation, data curation, writing – original draft, writing – review and editing. **Anna Clara Azevedo Silveira:** investigation, data curation, writing – original draft, writing – review and editing. **Cassiano Costa Rodrigues:** investigation, data curation, writing – original draft, writing – review and editing. **Bruna Cristina Borges:** investigation, data curation, writing – original draft, writing – review and editing. **Thaise Lara Teixeira:** investigation, data curation, writing – original draft, writing – review and editing. **Cecília Luiza Pereira:** investigation, data curation, writing – original draft, writing – review and editing. **João Paulo Silva Servato:** investigation, data curation, writing – original draft, writing – review and editing. **Normanda Souza Melo:** investigation, data curation, writing – original draft, writing – review and editing. All authors have read and agreed to the published version of the manuscript.

## Conflicts of Interest

None declared.

## Supporting information


**Supplementary Table 1**. Primers used to generate CRISPR/Cas9 *T. cruzi* P21^−/−^, confirmation of KO clones, RT‐PCR and qPCR.
