# Supplementary material for: Trypanosoma cruzi P21 Is a Pleiotropic Protein That Is Involved in Parasite Host Cell Invasion and Intracellular Parasitism
Source: Microbiologyopen. 2025 Nov 10;14(6):e70154. doi: 10.1002/mbo3.70154 (PMC12602995; doi:10.1002/mbo3.70154)
Supplement: Supplementary file 1 — Supplementary Table 1. Primers used to generate CRISPR/Cas9 T. cruzi P21−/−, confirmation of KO clones, RT‐PCR and qPCR. [file MBO3-14-e70154-s001.docx]

**Supplementary Table 1**. Primers used to generate CRISPR/Cas9 *T. cruzi* P21^-/-^, confirmation of KO clones, RT-PCR and qPCR.

| **Primers** | | |
| --- | --- | --- |
| **1** | sgRNA_36 Fw | GGAGGCCGGAGAATTGTAATACGACTCACTATAGGGAGAG**GCGCCTGCAGCGTGTCGGCCG**GTTTTAGAGCTAGAAATAGCAAG |
| **2** | sgRNA_424 Fw | GGAGGCCGGAGAATTGTAATACGACTCACTATAGGGAGAG**GTTCTACAAAGATACCGTGGT**GTTTTAGAGCTAGAAATAGCAAG |
| **3** | sgRNA_all genes Rv | CAGTGGATCCAAAAAAGCACCGACTCGGT |
| **4** | Bsd_ ultramer Fw | GTGTGAGAATAGGCTTTGTAAAAGGAATTTAATTTTACGGACACATCTCGCTAAACAGCAGCAACAACAGCAGGAGGAGC**ATGGCCAAGCCTTTGTCTCA** |
| **5** | Bsd_ultramer Rv | TCATTTTTCCATACAGTTGTCAGGCTGCCCCTTCTCTCCTCCTCTCCTGCAGCCGTGAAGAATCCCCCCATTCCGAGGTG**TTAGCCCTCCCACACATAAC** |
| **6** | Hygro_ ultramer Fw | GTGTGAGAATAGGCTTTGTAAAAGGAATTTAATTTTACGGACACATCTCGCTAAACAGCAGCAACAACAGCAGGAGGAGC**ATGAAAAAGCCTGAACTCAC** |
| **7** | Hygro_ ultramer Rv | TCATTTTTCCATACAGTTGTCAGGCTGCCCCTTCTCTCCTCCTCTCCTGCAGCCGTGAAGAATCCCCCCATTCCGAGGTG**CTATTCCTTTGCCCTCGGAC** |
| **8** | P21 Fw | GATACAACCACAAGGAGCC |
| **9** | P21 Rv | TTACTGGCGTCTGTGGAATC |
| **10** | UTR P21 Fw | GCCTCCATCCACATTTCATG |
| **11** | UTR P21 Rv | AACGTCCAATTAGGTCTTGTA |
| **12** | TcHGPRT Fw | CTACAAGGGAAAGGGTCTGC |
| **13** | TcHGPRT Rv | ACCGTAGCCAATCACAAAGG |
| **14** | TcMVK Fw | CGGCCGCGACATTTGGT |
| **15** | TcMVK Rv | GGCACTTCTAGGGCACGCAG |
| **16** | Diaz7 | CGCAAACAGATATTGACAGAG |
| **17** | Diaz8 | TGTTCACACACTGGACACCAA |
